# The Effects of Ambient Temperature on Lumbar Disc Herniation: A Retrospective Study

**DOI:** 10.3389/fmed.2022.811237

**Published:** 2022-07-19

**Authors:** Ping Wang, Cai Chen, Fanjie Liu, Fan Bu, Jianpeng An, Hao Qin, Qinghao Zhang, Tao Wang, Shengnan Cao, Wei Li, Bin Shi

**Affiliations:** ^1^State Key Laboratory of Precision Measurement Technology and Instruments, Tianjin University, Tianjin, China; ^2^Shandong Academy of Chinese Medicine, Jinan, China; ^3^Shandong Institute of Advanced Technology Chinese Academy of Sciences, Jinan, China; ^4^School of Control Science and Engineering, Biomedical Engineering Institute, Shandong University, Jinan, China; ^5^Bone Biomechanics Engineering Laboratory of Shandong Province, Shandong Medicinal Biotechnology Center, Neck-Shoulder and Lumbocrural Pain Hospital of Shandong First Medical University, Shandong First Medical University & Shandong Academy of Medical Sciences, Jinan, China

**Keywords:** ambient temperature, chronic disease, unhealthy habits, low back pain, lumbar disc herniation, distributed lag linear and non-linear models

## Abstract

**Purpose:**

This article was designed to provide critical evidence into the relationship between ambient temperature and intensity of back pain in people with lumbar disc herniation (LDH).

**Methods:**

Data concerning patient's age, gender, diagnostic logout, admission time, discharge time, residence area, and work area (residence area and work area were used to ensure research area) from 2017 to 2019 were obtained from the Neck-Shoulder and Lumbocrural Pain Hospital in Jinan, China. A total of 1,450 hospitalization records were collected in total. The distributed lag non-linear model (DLNM) was used to evaluate the relationship between lag–response and exposure to ambient temperature. Stratification was based on age and gender. Days 1, 5, 20, and 28 prior to admission were denoted as lags 0, 5, 20, and 28, respectively.

**Results:**

An average daily temperature of 15–23°C reduced the risk of hospitalization the most in men. Conversely, temperatures <10°C drastically increased hospitalization in men, particularly in lags 0–5 and lags 20–28. Men aged between 40 and 50 years old showed less effect in pain sensation during ambient temperature.

**Conclusion:**

High or low ambient temperature can increase the hospitalization risk of LDH, and sometimes, the temperature effect is delayed.

## Introduction

Low back pain (LBP) is a common disease among the middle-aged and elderly population that disrupts daily living (1, 2). It is often caused by muscle overuse and strain or repetitive injury sustained over a period of time. There are two types of LBP, namely, acute and chronic. Most cases that last for <3 months are considered acute; however, cases that persist for longer than 3 months are considered to be chronic. The prevalence of LBP generally increases with age. However, alarming reports show a growing trend of LBP in all age groups. It is reported that ~15–20% of adults experience back pain in a single year and 50–80% experience at least one episode of back pain in their lifetime (3). A survey among a university population in south China revealed that about 731/3,770 (19.39%) of all participants, particularly women, experienced chronic LBP (4). LBP combined with lower limb pain accounted for about 2/3 of patients who sought treatment for LBP in hospital, most of which was caused by lumbar disc herniation (LDH). Based on these statistics, LBP is increasingly recognized as a serious worldwide public health concern. LBP is prone to recurrence, and poor treatment effect will seriously affect patients' daily work and life, limit their participation in activities, and cause great burden on society and family (5). It is reported that people with LBP have an increased incidence of psychological morbidity. Bletzer et al. found that significantly different depression scores were reported for people with LBP compared to a healthy control group (6). In addition, LBP was shown to be strongly associated with insomnia (7) and produce neurological symptoms like radicular pain, radiculopathy, and lumbar spinal stenosis (8, 9). As a result, LBP has become one of the leading causes of disability in most countries and age groups (8). Worse yet, a 2018 study predicted that the upward trend of LBP prevalence and disability seen in the past 25 years will likely continue with the rise in the aging population (10).

It is, therefore, crucial to identify the causal factors for lower back pain. Previous studies have suggested some association between ambient temperature and the sensitivity and intensity of pain (11–13). A recent research conducted by Ziadé et al. found that pain was associated negatively with temperature, and a drop of 10°C in ambient temperature corresponded to an increase of 0.5 points on the pain numerical scale, which means temperature could explain 22% of pain variance (14). In contrast, related molecular biology experiments also revealed there is a relationship between ambient temperature and pain. The transient receptor potential (TRP) channel, ubiquitous in all cells (15, 16), regulates the permeability of cations like Ca^2+^ and Na^+^ and mediates sensory signal transmission, including that of temperature and pain (17). The TRP family members TPRV1, TPRV3, TPRV4, TRPM8, and TRPA1 are widely expressed in the dorsal root ganglion and skin where the sensations of temperature and pain are received and translated into physiological signals (18–20).

Therefore, given the high incidence of LBP and the harm it causes, it is important to figure out the risk factors for its acute exacerbation. The primary purpose of this article was to determine the working relationship between temperature and intensity of back pain in people with LDH in Jinan, China.

## Method

**Figure 1:**
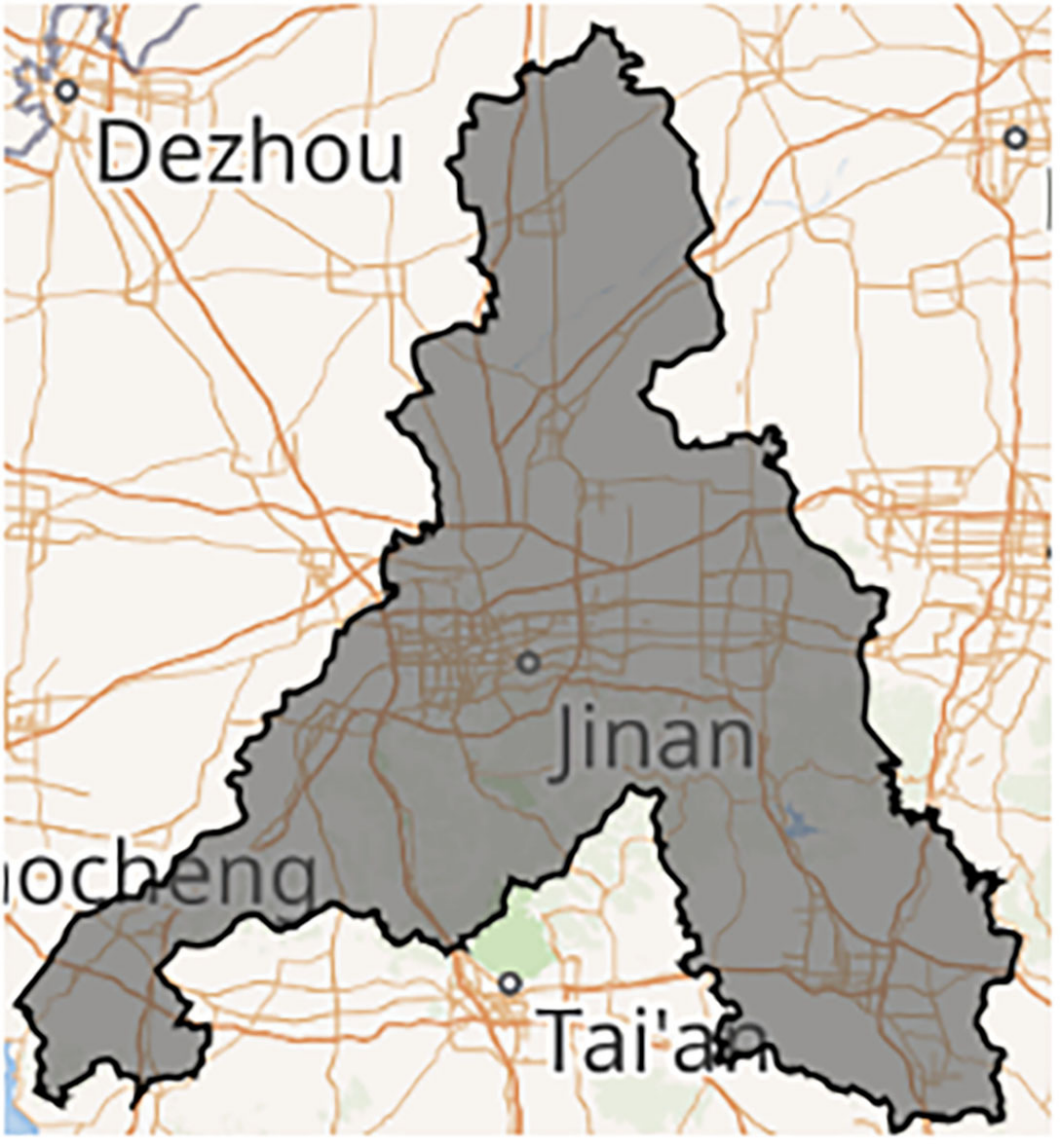
Location of research area.

**Figure 2:**
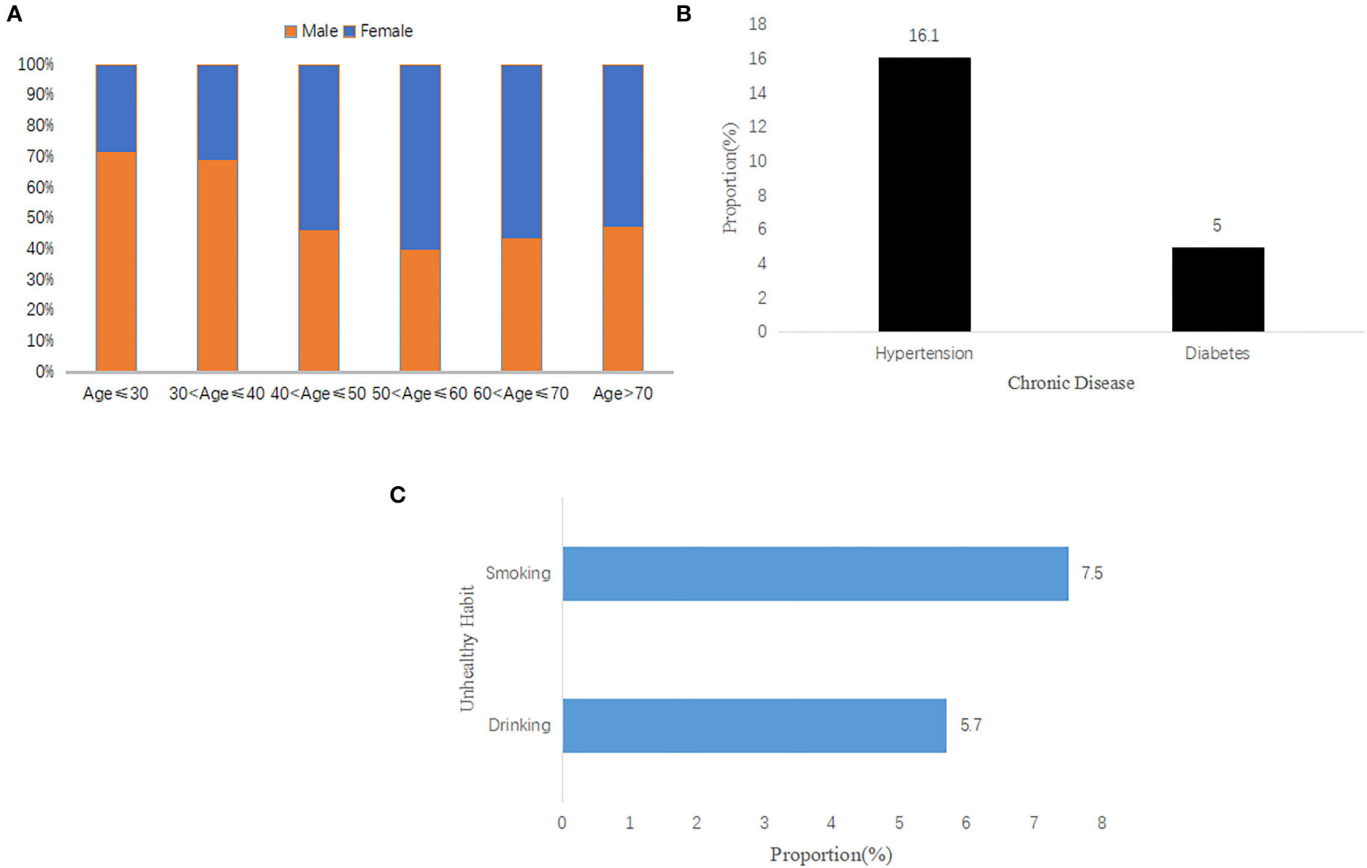
Descriptive results about hospitalization records. **(A)** The proportion of gender distribution among different age groups; **(B)** the proportion of chronic disease among whole amount; **(C)** the proportion of unhealthy habits among whole amount.

The following information, from the year 2017 to 2019, was collected from the Neck-Shoulder and Lumbocrural Pain Hospital in Jinan, China: patient's age, gender, diagnostic logout, admission time, discharge time, residence area, and work area (residence area and work area were used to ensure research area). A total of 3,550 records were collected in total. The data inclusion criteria were (1) people residing in Jinan (Figure 1) and (2) people diagnosed with LDH. After critical review, 1,450 hospitalization records on patients with LDH were used for the study. Temperature data from the same period were obtained from the Shandong Environmental Protection Department.

Distributed lag non-linear model (DLNM), a widely employed model used to evaluate the relationship between lag–response and exposure to ambient temperature, was used in this study. It describes the associations by plotting potentially non-linear and delayed effects in a time series (21–23). The DLNM is presented as follows:


(1) $$\begin{array}{l} \begin{array}{ccc} \text{Log}[\text{E}(\text{y})] & = & \alpha +\text{Temperature}+\text{ns}(\text{time, df})+\text{ns}(\text{Humidity, df}) \end{array}\\ \;\begin{array}{cc} + & \text{factor}(\text{DOW})+\text{factor}(\text{Holiday}) \end{array} \end{array}$$

where Log[.] is the connected function, E(y) is the number of hospitalizations, α is the constant term, Temperature is the daily average temperature, time is the time variable, ns(.) is the natural cubic splines, df is the freedom of degree (time, 2; humidity, 3), DOW is the day of the week, and Holiday is the holiday variable. Previous studies have demonstrated a lag effect of temperature on people's health (24, 25). For this study, a lag effect of daily average temperature was considered. For convenience of expression, the day of patient admission was named as lag 0, and the first day before admission was denoted as lag 1; similarly, the 28th day before admission was denoted as lag 28. Stratification was based on age and gender. All the statistical analyses mentioned above were conducted using the *RStudio* software.

## Result

### Descriptive Analysis Results

The daily average temperature and relative humidity in Jinan between the years 2017 and 2019 are summarized in Table 1. The maximum and minimum daily average temperatures were 33.4 and −9.1°C, respectively. The study had a near equal number of men (747 or 51.5%) and women (703 or 48.5%). The age distribution was as follows: age < 30 = 7%, 30 ≤ age < 40 = 13%, 40 ≤ age < 50 = 23.4%, 50 ≤ age < 60 = 26.3%, 60 ≤ age < 70 = 19.9%, and age ≥ 70 = 10.7%, respectively. The maximum and minimum relative humidity in Jinan between 2017 and 2019 were 14 and 98, respectively.

**Table 1:** The daily average temperature and relative humidity.

	**Min**	**P_**25**_**	**P_**50**_**	**P_**75**_**	**Max**
Temperature	−9.1	6	16.5	25.3	33.4
Humidity	14	36	48	64.75	98

*Min, minimum; max, maximum; P25, the first quartile; P50, the second quartile; P75, the third quartile*.

The percentage bar chart based on age and gender is shown in Figure 2A. The age group age ≤ 30 and 30 < age ≤ 40 comprised more men than women, whereas the contrary was true among the age groups 40 < age ≤ 50, 50 < age ≤ 60, 60 < age ≤ 70, and age > 70. As can be seen from Figure 2B, the proportion of hypertension and diabetes are 16.1 and 5%, respectively. Figure 2C describes the proportion of smoking and drinking, which are 7.5 and 5.7%, respectively.

### DLNM Result

**Figure 3:**
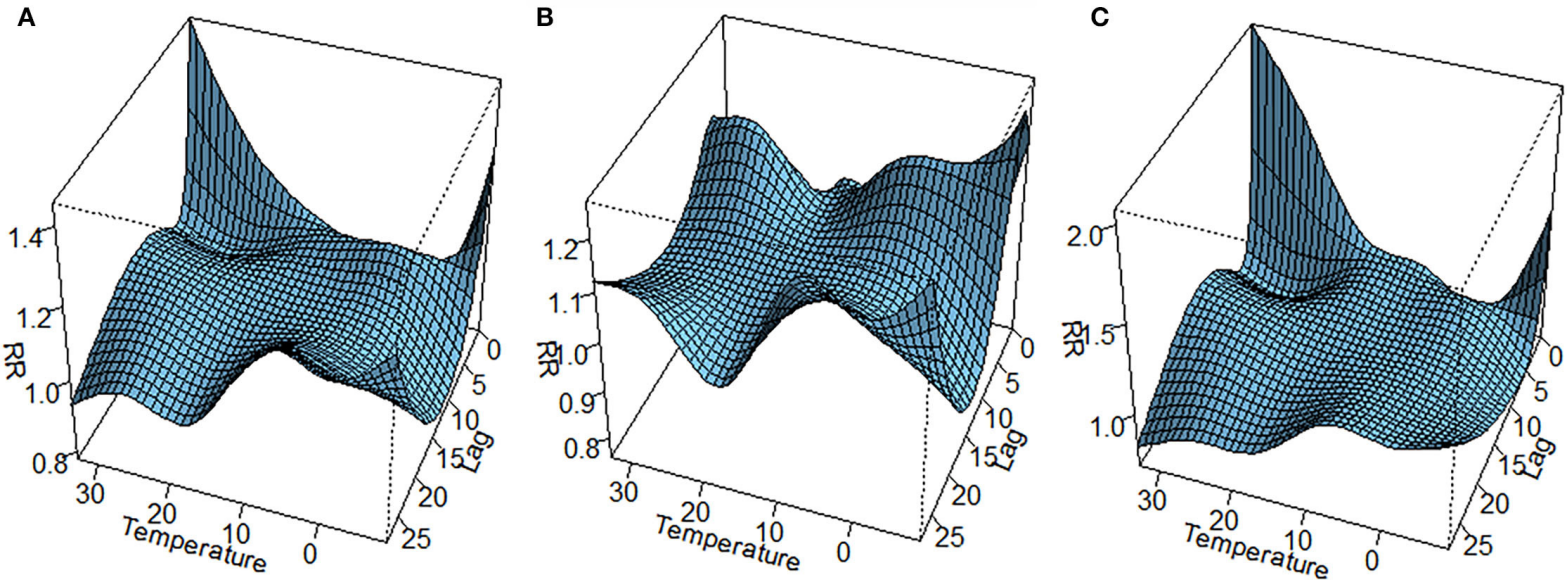
Three-dimensional lag–response curve of temperature [**(A)** whole; **(B)** men; **(C)** women]. RR, relative risk.

**Figure 4:**
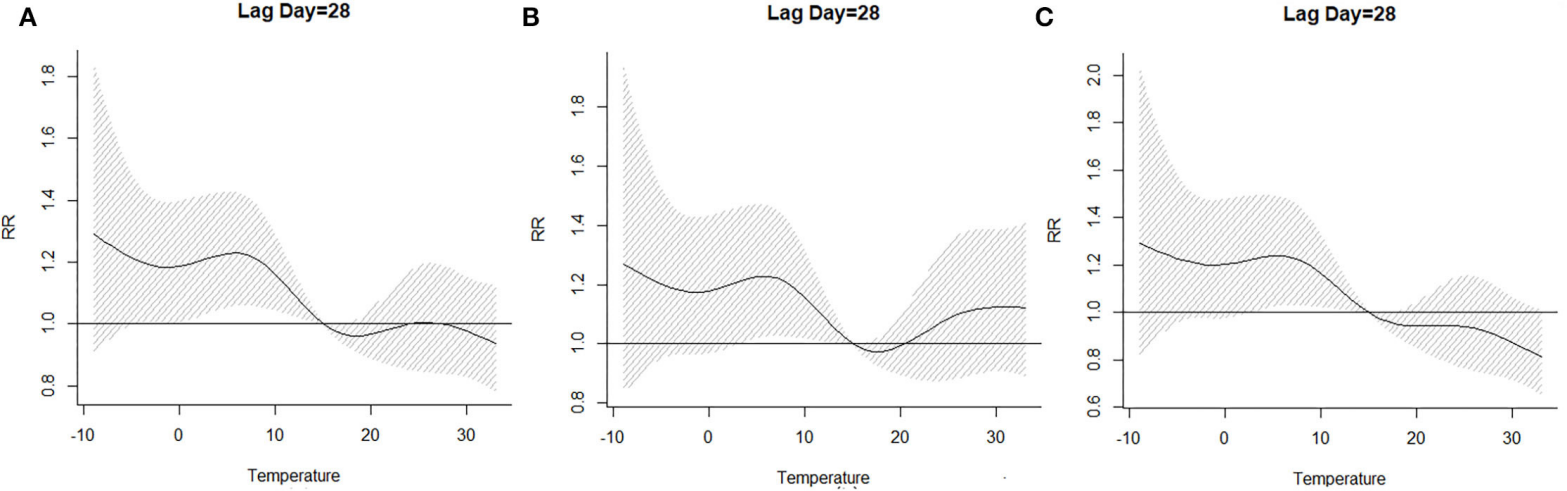
The effect of temperature on the hospitalization when lag day was on 28 [**(A)** whole; **(B)** men; **(C)** women]. RR, relative risk.

**Figure 5:**
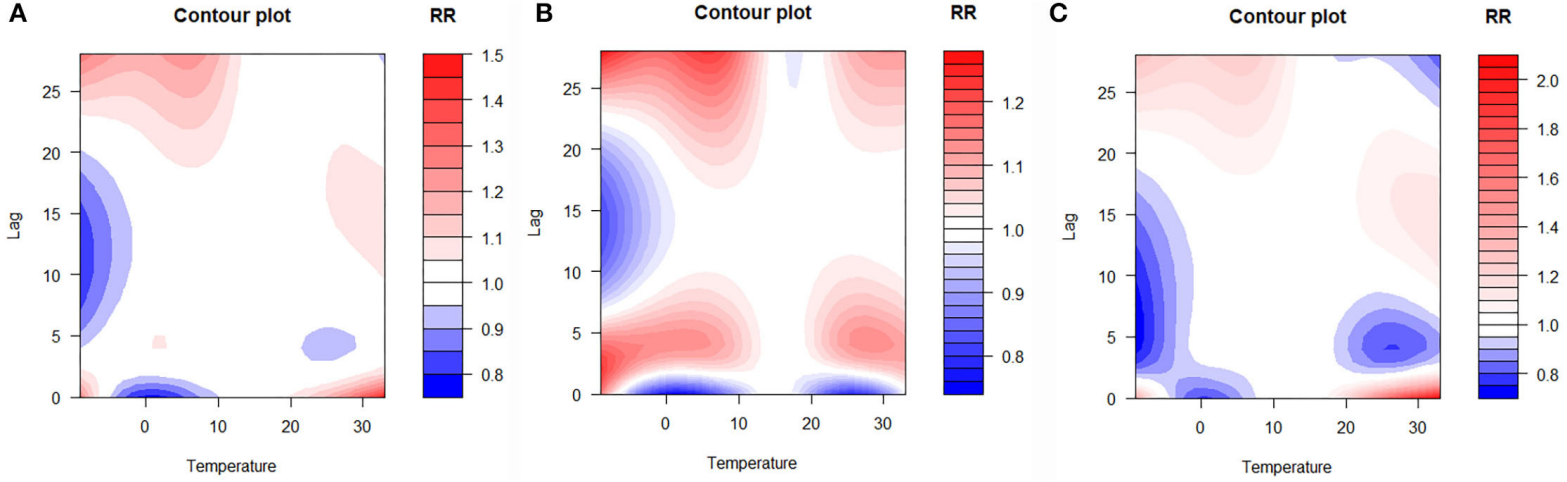
Contour plot of lag–response about temperature on hospitalization [**(A)** whole; **(B)** men; **(C)** women]. RR, relative risk.

The lag–response curve of temperatures between the years 2017 and 2019 is shown in Figure 5. As is evident in Figure 3, the male vs. female response to 25–30°C temperature was markedly different, which was that women were more sensitive to higher temperature on lags 0–5 than men in that conditions. In addition, the risk for men with lower temperature was greater than women. Figure 4 illustrates the effect of temperature on hospitalization of the patients with LDH and back pain. A daily average temperature of 15–23°C provided the lowest risk of hospitalization in men, while in women, temperatures >15°C garnered the lowest risk. The contour plot of lag–response to temperature revealed that temperature <10°C gradually increased the risk of male hospitalization, especially during lags 0–5 and lags 20–28. Similarly, temperature >21°C also produced a lag effect that exacerbated the risk of hospitalization (Figure 5).

**Figure 6:**
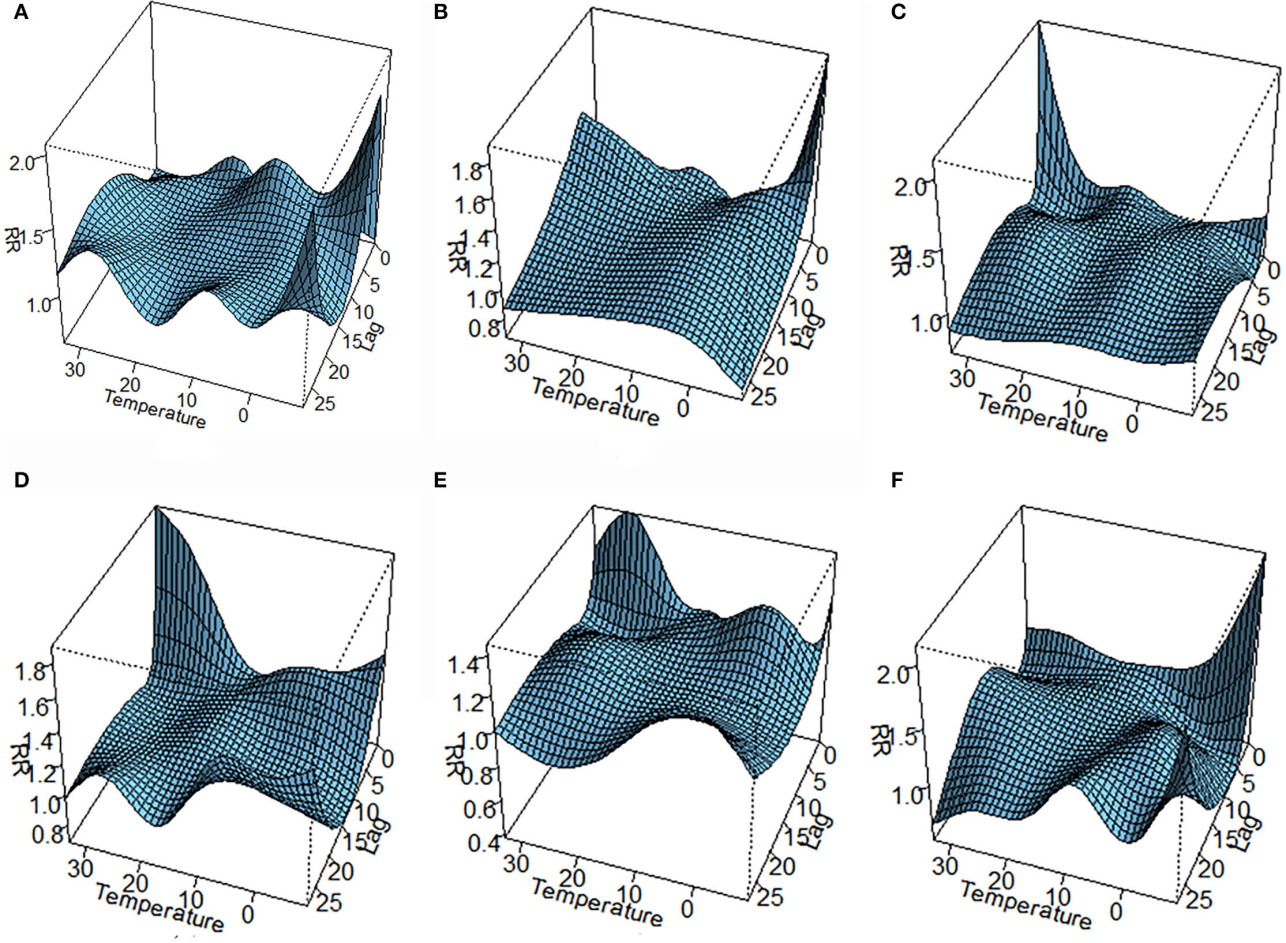
The three-dimensional lag–response to temperature based on stratification of age [**(A)** age ≤ 30 group; **(B)** 30 < age ≤ 40 group; **(C)** 40 < age ≤ 50; **(D)** 50 < age ≤ 60; **(E)** 60 < age ≤ 70; **(F)** age > 70].

**Figure 7:**
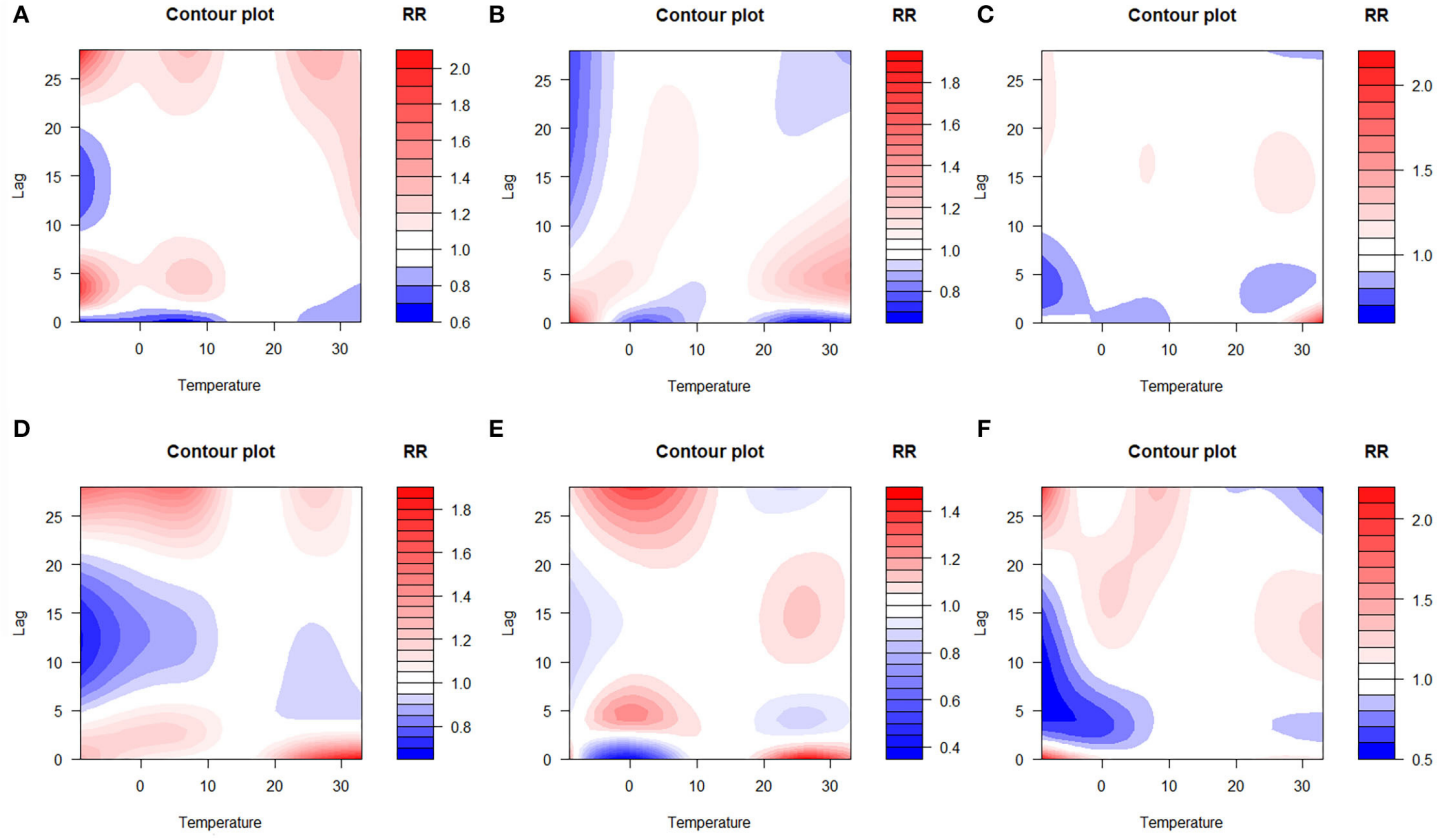
Contour plots of lag–response to temperature based on the age stratification [**(A)** age ≤ 30 group; **(B)** 30 < age ≤ 40 group; **(C)** 40 < age ≤ 50; **(D)** 50 < age ≤ 60; **(E)** 60 < age ≤ 70; **(F)** age > 70].

**Figure 8:**
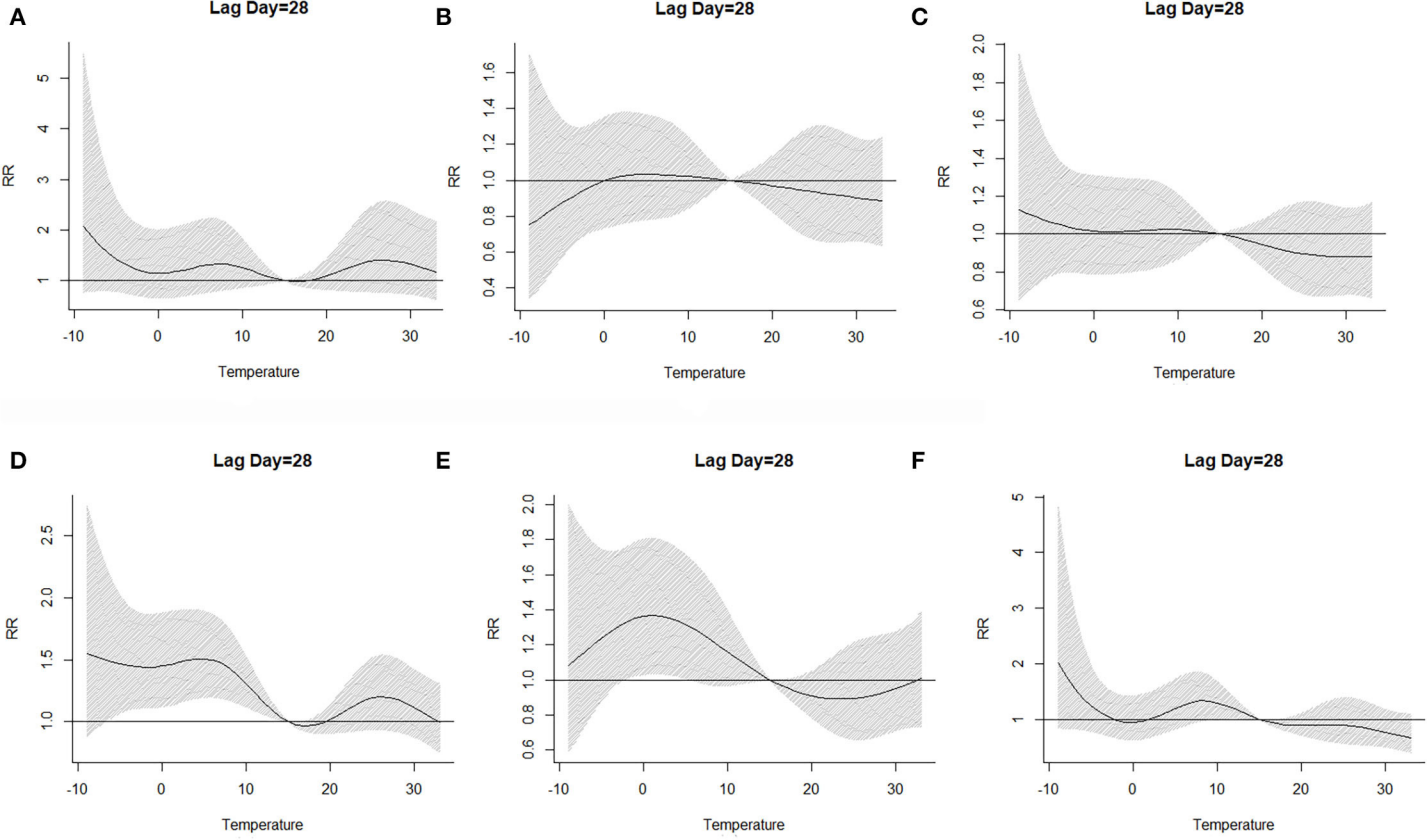
The effect of temperature on the hospitalization when lag day was on 28 [**(A)** age ≤ 30 group; **(B)** 30 < age ≤ 40 group; **(C)** 40 < age ≤ 50; **(D)** 50 < age ≤ 60; **(E)** 60 < age ≤ 70; **(F)** age > 70].

The three-dimensional plot of lag–response to temperature based on the stratification of age is provided in Figure 6. Interestingly, our analysis demonstrated that the 40 < age ≤ 50 age group was not markedly affected by ambient temperature, and Figure 8C also confirmed this point. Contour plots of lag–response to temperature based on the age stratification are described in Figure 7. It can be found that both higher and lower temperature can increase the risk of hospitalization, which means that temperature had an adverse influence on the LDH with back pain. Finally, the effect of temperature on hospitalization on lag 28 is shown in Figure 8. It is apparent from this figure that these groups (50 < age ≤ 60 and 60 < age ≤ 70) are more affected by low temperature.

## Discussion

LDH, characterized by an early symptom of LBP, can seriously reduce one's quality of life (7, 26–29). The purpose of this article was to investigate whether ambient temperature worsened the symptoms of LBP in patients with LDH.

Based on our results, ambient temperature increased the risk of hospitalization in patients with LDH, which proved our hypothesis that pain perception in patients with LDH is exacerbated in both low and high ambient temperatures. Previous studies have demonstrated a link between ambient temperature and the sensitivity and intensity of pain (11, 12, 30). Hedelin et al. suggested that ambient temperature can alter the threshold of pain in patients with chronic pelvic pain syndrome. One possible mechanism for this phenomenon could be the sensitization of the nociceptive C-fibers, responsible for hot/cold sensation, to innocuous temperature gradients due to the activation of the TRP ion channels.

Furthermore, a high-temperature environment can relax peripheral blood vessels and increase blood circulation to the periphery in an effort to cool the body. Patients with LDH have microcirculation disorders (31), which may prevent the cooling mechanism and contribute to an increased sensation of pain. On the contrary, a low-temperature environment constricts peripheral blood vessels in an effort to maintain body warmth. During blood vessel constriction, blood components like fat, sugar, and fibrinogen tend to accumulate in the vessels and accentuate inflammatory response at the site of protrusion. Under these circumstances, blood viscosity, plasma viscosity, erythrocyte specific volume, and erythrocyte aggregation index were shown to rise significantly in patients with LDH, as opposed to the healthy population (32, 33). Similar reports were demonstrated in healthy rats exposed to high temperature; i.e., they exhibited a large increase in whole blood viscosity, plasma viscosity, and erythrocyte aggregation index (34). Furthermore, it was shown that ambient temperature was negatively associated with blood flow velocity and vasoreactivity and positively associated with cerebrovascular resistance (35). Based on these results, it is evident that ambient temperature may change hemorheological factors, and thereby exacerbating pain symptoms.

However, some studies have demonstrated conflicting results (12, 36, 37). Possible reasons behind these conflicting reports may be (1) failure to address the delayed effect as these studies only used a simple correlation to evaluate the temperature and pain relationship; (2) possible existence of noise that was not removed; and (3) differences in the location of study, climate, and medical advancement.

### Unhealthy Habits and Pain

Our findings revealed that 5.9 and 7.8% of the patients with LBP were heavy drinkers and smokers, respectively. These reports are in accordance with prior studies that showed that patients with smoking and drinking abuse had a relative risk of 4.489 and 3.326 of LBP, respectively, compared to patients without these risk factors (38). The Shemory et al. report was further corroborated by other studies that demonstrate a significant relationship between alcohol or tobacco use and LBP (29, 39, 40).

### Chronic Diseases and Pain

Our results disclosed that ~50% of the patients with LDH were also diagnosed with diabetes (Figure 2). This is in accordance with previous reports of a high prevalence of LBP (37.1 vs. 30.3%) among diabetics and a 1.20 (95% CI 1.06–1.35) high association between diabetes and risk of LBP (41). This was further corroborated in another study that showed ~6% of patients with LBH developed diabetes (42, 43). In a separate study, multiple other diseases and lifestyle factors were shown to increase the incidence of recurring non-specific LBP: a sedentary lifestyle caused a 3.5-fold increase (*p* <0.001), excessive coffee consumption spiked LBP risk (OR = 16.44, 95% CI 8.55–31.61), and cigarette smoking increased the likelihood of both recurrent and chronic LBP. Metabolic syndromes like high blood pressure (over 9-fold), type 2 diabetes (over 3-fold), and hyperlipidemia (over 2-fold) (*p* < 0.001, <0.001, and <0.01, respectively), were also shown to markedly elevate chronic LBP risk (44). In our study, 16% of the patients with hypertension also had LBP. This result hints at a long suggested association between hypertension and the prevalence of LBP (45, 46).

The limitations of this study were as follows. (1) The potential effects of medicines on LDH were not considered. Given that a good portion of patients with LDH also experienced chronic diseases, it is possible that medicinal effects could have contributed to LBP. (2) The occupation (the nature of which could contribute to LBP) of subjects in this study was not considered. (3) The data about height and weight were not contained in hospitalization records, and as a result, the body mass index was not involved in our study. Our future goal is to include these factors and perform an extensive study into the seasonal effects of temperature and relative humidity on LBP.

## Conclusion

Higher or lower ambient temperature could increase the hospitalization risk of LDH, and there was a delay effect. In contrast, unhealthy habits and chronic diseases also can lead to a sharp increase in LBP hospitalizations.

## Data Availability Statement

The original contributions presented in the study are included in the article/supplementary material, further inquiries can be directed to the corresponding author/s.

## Ethics Statement

Written informed consent was obtained from the individual(s) for the publication of any potentially identifiable images or data included in this article.

## Author Contributions

PW, WL, and BS: guarantor of integrity of entire study and study concept. CC: study design. FL and PW: literature research. FB, JA, and HQ: data acquisition. CC and PW: data analysis. PW: manuscript preparation. WL and BS: manuscript editing. CC, PW, and WL: manuscript revision/review. All authors have participated in this study and consent to publish this article in journal. All authors contributed to the article and approved the submitted version.

## Funding

This study was supported in part by grants from National Key R&D Program of China (2019YFE0117800), major scientific and technological innovation projects of Shandong Province (2019JZZY020904, 2021CXGC010506, and 2018CXGC1310), Shandong Institute of Advanced Technology Chinese Academy of Sciences (#YJZX003), Science and Technology Development Program of Shandong Academy of Medical Sciences (Grant No. 2018-23), the Natural Foundation of Shandong Provincial Natural Science Foundation (ZR2019MH134, ZR2021QH290, ZR2020MH386, and ZR2019MH134), Collaborative Innovation Center Project of quality Control of traditional Chinese Medicine and Construction of whole Industry chain in Colleges and Universities of Shandong Province (CYLXTCX2020-04 and CYLXTCX2021-01); special project for prevention and control of pneumonia caused by novel coronavirus in Jinan (202001006), the Academic Promotion Project of Shandong First Medical University (Grant No. 2019QL003), and the Shandong Provincial Central Government Guides Local Science and Technology Development Fund Projects (YDZX20203700002055), National Natural Science Foundation of China (22176115), and National Training Program for Innovative Backbone Talents of Traditional Chinese Medicine.

## Conflict of Interest

The authors declare that the research was conducted in the absence of any commercial or financial relationships that could be construed as a potential conflict of interest.

## Publisher's Note

All claims expressed in this article are solely those of the authors and do not necessarily represent those of their affiliated organizations, or those of the publisher, the editors and the reviewers. Any product that may be evaluated in this article, or claim that may be made by its manufacturer, is not guaranteed or endorsed by the publisher.

## Abbreviations

LDH: lumbar disc herniation
DLNM: distributed lag non-linear model
LBP: low back pain
DOW: day of week
TRP: transient receptor potential.
